# The antecedents of customer satisfaction in the live-streaming commerce of green agricultural products

**DOI:** 10.1371/journal.pone.0305527

**Published:** 2024-07-12

**Authors:** Ying Wang, Lin Fang, Jialing Pan

**Affiliations:** School of Business, Wenzhou University, Wenzhou, China; Shandong University, CHINA

## Abstract

Live-streaming technology has been widely adopted to promote the sale of green agricultural products. Based on the literature regarding electronic commerce and customer satisfaction, this article integrates expectation-disconfirmation theory and the SERVQUAL model to investigate the antecedents of customer satisfaction and the routes along which the former drives the latter in the live-streaming commerce of green agricultural products. Our results demonstrate that most consumers are satisfied with the live-streaming commerce of green agricultural products, with an overall satisfaction degree of medium to high. In addition, a total of four antecedents are identified, namely commodity, live-streaming platforms, live-streaming contents and supporting services. Among the variables relevant to commodity, “commodity brand building” has the highest weight. Meanwhile, the corresponding variables for live-streaming platforms, live-streaming contents and supporting services are “interface design”, “live-streaming atmosphere” and “privacy protection”, respectively. Furthermore, live-streaming platforms are found to have the strongest direct influence on customer satisfaction, while commodity is found to have the strongest indirect and total influence on customer satisfaction. The theoretical and managerial implications are discussed at the conclusion of this article.

## Introduction

Sustainable development is the real need and unavoidable selection of human development in the future and food safety is pertinent to the survival of human society [1]. In recent years, there has been growing concern about the deterioration of the natural environment and the health of human beings. In this context, green agricultural products, characterized as resource-conserving and environmentally friendly, are increasingly favored by consumers. Due to the lower prices, greater convenience, time savings and better product and service choices that e-commerce provides, more and more consumers are shifting their buying activities from bricks-and-mortar stores to online shopping platforms. The contribution of the Internet to e-commerce has been fundamental, opening up new channels of communication and commerce and enabling consumers to obtain products and services at the touch of a button [2]. As a new Internet trend, live video streaming with high interactivity shows current real-time situations to end users via the Internet and has been tremendously popular [3]. Consequently, live-streaming has been adopted in e-commerce to show how products are created and used, to display different perspectives of products, to respond to consumers’ questions in real time and to arrange live events which entertain and encourage consumers to buy on the spot. At present, there are more than 30,000 agricultural e-commerce platforms in China, among which 3000 are e-commerce platforms of green agricultural products, with rural e-commerce far from being saturated [4]. Clearly, it is necessary to employ live-streaming to boost the development of green agricultural product e-commerce. Given that satisfaction is crucial to the realization of long-term objectives and is directly related to the success of a company [5], discerning the antecedents of customer satisfaction in the live-streaming commerce of green agricultural products is of great significance for achieving sustainable growth in the industry.

Satisfaction is often described as the attitudinal manifestation of a consumer’s overall evaluation of one or more consumption periods. Specifically, customer satisfaction is defined as the outcome of the interplay between pre-purchase expectations and post-purchase evaluations [6]. When satisfied, i.e., post-purchase evaluations are higher than pre-purchase expectations, consumers are more willing to share their favorable experience with others and provide positive word-of-mouth recommendations [7]. On the other hand, companies with satisfied customers can benefit from positive word of mouth [8] and greater customer loyalty (e.g., [9, 10]). Therefore, customer satisfaction is perceived as the precondition of customer loyalty.

Extant literature on customer satisfaction can be categorized into three distinct streams. The first stream, which earlier studies belong to, concentrated on customer satisfaction as a post-consumption mental state. The majority of these investigations are in consumer psychology and behavior, aiming to delineate the nature of customer satisfaction and unravel the cognitive processes underlying its emergence (e.g., [11–14]). The second stream focuses on the consequences of customer satisfaction, such as repurchase intention (e.g., [15, 16]), consumer spending [17] and loyalty (e.g., [10, 18–20]).

The third stream examines the antecedents of customer satisfaction. Prior studies have discerned a broad range of factors which affect customer satisfaction in either the offline shopping environment or the context of e-commerce. While advertising (e.g., [21]) and product variety (e.g., [22]) are found to influence customer satisfaction, the four dimensions of e-shopping quality, i.e. customer service, website design, atmosphere and privacy, are discovered as the determinants of the perceptions of enjoyment, which in turn influence consumers’ attitudes toward e-shopping, e.g., satisfaction [23]. Besides, some scholars identified brand as a crucial determinant of customer satisfaction (e.g., [24, 25]), whereas others demonstrated that logistics service, such as communication of delivery status, convenience of receipt, reception experience and convenience of return, has a significant positive impact on customer satisfaction (e.g., [26, 27]). Furthermore, price, easiness of return, packaging and product information are discerned as the determinants of customer satisfaction with Amazon.com [28]. Characterized by better user experience and stronger interaction, live-streaming commerce has emerged as a new trend of social commerce and favored by more and more consumers. Consequently, there has been growing interest in exploring the antecedents of customer satisfaction in live-streaming commerce. On the one hand, content types significantly influence psychological and social needs, e.g., the affective and social integrative satisfaction of viewers’ rose when watching beauty influencers [29]. On the other hand, anchor characteristics of influence, sales promotion and interactive entertainment positively affect consumers’ purchasing behavior, the prerequisite of which is satisfaction, in live-streaming commerce [30].

With regard to the theoretical underpinning, expectation-disconfirmation theory (EDT) and the SERVQUAL model are widely used to explore customer satisfaction in different disciplines [31]. First proposed by Oliver in 1977, ETD describes satisfaction as the discrepancy between customers’ pre-purchase expectations and post-purchase perceived performance. Disconfirmation is positive if the perceived performance is greater than pre-purchase expectations, yielding satisfaction, whereas disconfirmation is negative if the perceived performance is smaller than pre-purchase expectations, yielding dissatisfaction [12]. According to Parasuraman et al. (1985) [32], the SERVQUAL model gauges service quality by the difference between consumers’ expectations and their perceived performance. SERVQUAL is composed of five dimensions, i.e. tangibility, reliability, empathy, responsiveness and assurance. Many scholars have used SERVQUAL to demonstrate the positive significant influence of service quality on customer satisfaction in diverse areas (e.g., [31, 33–35]).

However, most of prior studies consider single or several antecedents of customer satisfaction instead of from a holistic perspective. In addition, past studies have primarily focused on traditional e-commerce, with little attention paid to live-streaming commerce, in spite of the fact that the antecedents of customer satisfaction differ across purchasing channels. Besides, existing research is e underpinned by a single theory or uses the theories separately [36]. To fill these gaps, this article intends to make a preliminary attempt to holistically examine the antecedents of customer satisfaction in live-streaming commerce of green agricultural products, by virtue of integrating the widely used EDT and the SERVQUAL model. In doing so, we provide empirical evidence for the validity of the SERVQUAL model in live-streaming commerce of green agricultural products, reveal the mechanism of how customer satisfaction is driven in this context and provide actionable guidance to managers to better foster customer satisfaction and loyalty in this industry.

The remainder of this article is organized as follows: Section 2 reviews the literature associated with electronic commerce and customer satisfaction; Section 3 primarily introduces the research methodology; Section 4 presents the results of data analysis; Section 5 discusses the main findings of our research and Section 6 concludes the study and elaborates the implications for both the academia and industry.

## Literature review

### Electronic commence

E-commerce is defined as the process of buying, selling or exchanging products, services and information over a computer network, e.g., the Internet [37]. With wide prospects for development, e-commerce leverages advancements in internet technology to integrate with the economy, resulting in expanded transaction scales. Characterized by multiple virtues such as cost reduction, extended distribution channels and business model diversification, e-commerce is a complex IT process supporting the overall customer experience from design, communication, delivery, implementation and evaluation [38]. The intense global competition among e-commerce merchants necessitates a focus on enhancing the general quality of online retailers for customer satisfaction in business-to-consumer (B2C) e-commerce (e.g., [39, 40]). Therefore, how to "stand out" among the various competitors to obtain the loyalty of more consumers is of great significance to merchants on the e-commerce platforms. Obviously, current e-commerce research should be consumer-oriented, paying closer attention to consumers’ actual feelings during their online shopping.

Up to now, there has been a vast array of literature exploring consumer issues in e-commerce, among which purchase intentions have received the lion’s share of attention. Many of these studies have focused on the factors determining consumers’ purchase intentions from different perspectives, e.g., product and platform characteristics (e.g., [41]), interaction (e.g., [42]), trust (e.g., [43, 44]), web interface (e.g., [45, 46]) and online reviews (e.g., [47, 48]). With the prevalence of e-commerce, green agricultural products, favored by consumers for their safety and environmental friendliness, have also promoted the sales online. However, the volume of academic attention paid to consumer issues in the e-commerce of green agricultural products is scant.

### Social commerce and live streaming

Social commerce, or s-commerce, is a branch of e-commerce, which promotes social interaction by virtue of social media to facilitate online transactions and reinforce the online shopping experience (e.g., [49, 50]). The main reason consumers use social commerce is to find more product information and to take action on their purchase decisions. Consequently, many scholars have studied the media through which social commerce stimulates consumers’ consumption behavior, such as web quality [51], social commerce feature richness [46], informational support [52] and interaction with sellers [53].

With the further development of social commerce, live-streaming commerce has become an emerging model. By using one or more types of communication technology, images and sounds can be instantly transmitted from one place to another, making the viewers of live-streaming commerce feel like they are present at the event. To facilitate consumer purchase decisions, a growing number of firms are using live-streaming services as a sustainable marketing communication channel to reach existing and potential consumers [54]. As content creators, live streamers play a significant role in live streaming. Specifically, the more appealing their content is to consumers, the more attention and influence they will attain [55]. Undoubtedly, live streaming is an innovative and effective way to promote the sales of e-commerce. However, research into live streaming is still at a nascent stage, with most studies depicting the characteristics of live streaming and consumers’ motivations to participate in it [56].

### Customer satisfaction

In prior studies, scholars have defined customer satisfaction from different viewpoints. From the perspective of companies, customer satisfaction has been interpreted as a market-based asset which is associated with the efficient and effective coordination of company resources and the strengthened company performance (e.g., [56–58]). The crucial role of customer satisfaction as an indicator of a company’s performance has been identified and its relationship with market share and the financial results of the company is well established [59]. From the perspective of consumers, customer satisfaction refers to a consumer’s subjective assessment of any result or experience related to purchasing a product [60]. When they are satisfied, consumers are inclined to inform others of their excellent experience and make positive word-of-mouth recommendations. Given the specific objective of this study, we define customer satisfaction as consumers’ evaluation of their shopping experience in the live-streaming commerce of green agricultural products.

Customer satisfaction has been widely explored using expectation-disconfirmation theory (EDT) in multiple disciplines, such as marketing, psychology and medicine [31]. According to EDT which is first proposed by Oliver (1977), customer satisfaction may be indicated by the difference between the perceived performance and customer expectations. Specifically, positive disconfirmation comes into being if the perceived performance exceeds the initial expectation, resulting in satisfaction, whereas negative disconfirmation comes into being if the perceived performance falls behind the initial expectation, resulting in dissatisfaction [12]. Founded on prior studies, Parasuraman et al. (1985) [32] established a conceptual model of service quality, namely the SERVQUAL model, by regarding service quality as the difference between customers’ initial expectations and their actual perceived performance. As a result of repetitive refinements, SERVQUAL focuses on five dimensions of service quality: tangibles (physical facilities, equipment, and appearance of personnel), reliability (ability to perform the promised service dependably and accurately), responsiveness (willingness to help customers and provide prompt service), assurance (knowledge and courtesy of employees and their ability to inspire trust and confidence), empathy (caring, individualized attention the firm provides its customers). Since its introduction, a host of scholars have tested the SERVQUAL model and developed scales to precisely gauge service quality. With the application and validation in multiple disciplines, SERVQUAL has been widely used as an instrument to explore customer satisfaction in both academia and practice (e.g., [31, 34, 61, 62]).

Prior studies have widely acknowledged that customer satisfaction is a crucial determinant of future purchase (e.g., [63]) and a key driver for loyalty (e.g., [64–66]). Therefore, there has been a vast literature in marketing and economics on the antecedents of customer satisfaction (e.g., [2, 9, 67, 68]). Early studies mainly focus on the offline setting and the corresponding factors influencing customer satisfaction include the psychological environment of the store, the diverse procedures customers have to follow (e.g., queues, cashiers and trolleys), the moments of contact with people and the retailer’s key offer, namely product quality, variety, assortment and pricing policy [22]. In addition to product pricing, packaging also has a statistically significant influence on customer satisfaction [69]. Besides, both rational brand quality, e.g., product quality, service quality, and distribution quality, and emotional brand associations, e.g., advertising style, brand image and salesperson’s personality, positively affect customer satisfaction [24].

Due to the prosperity of e-commerce, the volume of online consumers is increasingly growing and e-customer satisfaction has been a particularly interesting aspect of this. Some scholars argue that e-customer satisfaction is determined by website features, such as website aesthetics (e.g., [70, 71]), website size and website quality (e.g., [72]). The image of a website involves its design, which is intended to increase website quality and to satisfy customers in the e-commerce environment (e.g., [23, 73]). While brand identity and brand image are identified as crucial determinants of customer satisfaction and purchase intention in the digital era [25], price is found to possess divergent impact on sales and customer satisfaction in the context of content sharing on the Internet [74]. To be specific, higher price leads to higher sales and lower customer satisfaction for certified sharers, while higher price leads to lower sales and higher customer satisfaction for non-certified sharers. Meanwhile, logistics (e.g., [26, 27]), privacy (e.g., [23, 73]), information quality (e.g., [28, 71]), atmosphere (e.g., [23]), customer service (e.g., [23, 75]) and advertising (e.g., [21]) are also identified as the factors that drive e-customer satisfaction. With the continuous development of information technology, social commerce has been prevailing due to its fundamental features, such as user interactions and user-generated content (e.g., [76]). As a result of its better user experience and stronger interaction with consumers, live-streaming commerce has become the new trend of social commerce. Although the volume of scholarly work is still scarce, scholars have started to examine customer satisfaction in the context of live-streaming, especially those unique issues which do not emerge in the bricks-and-mortar stores or other e-commerce formats. For instance, anchor characteristics of influence, sales promotion, and interactive entertainment are found to positively impact consumers’ purchasing behavior, the prerequisite of which is satisfaction, in live-streaming commerce [30], whereas different content types of live-streaming commerce differed significantly in satisfying psychological and social needs, in particular, viewers’ affective and social integrative satisfaction increased when watching beauty influencers [29].

In short, extant literature mainly focused on single or several factors affecting customer satisfaction without a holistic perspective. In addition, most of the relevant studies have been conducted in the context of traditional e-commerce and little attention has been paid to live-streaming commerce. Moreover, regarding the theoretical underpinning, the majority of prior studies focused on a single theory or used the theories separately [36]. To address these gaps, this study makes a preliminary attempt to holistically explore the determinants of customer satisfaction in live-streaming commerce of green agricultural products, a seldom tapped topic in prior studies, based on the integration and implication of EDT and the SERVQUAL model.

## Methodology

### Data collection

This study employed an online anonymous survey to collect data. The questionnaire consisted of two parts. Part 1 collected consumers’ basic information, i.e. demographic and socio-economic characteristics and shopping experience in the live-streaming commerce of green agricultural products. Part 2 collected information on customer satisfaction with the live-streaming commerce of green agricultural products via 18 items, among which one measured customers’ overall satisfaction and 17 investigated the potential factors influencing customer satisfaction (see **Table 1**). Following Kuksov and Xie [77] we employed respondents’ ratings to measure their satisfaction for all the 18 items, each of which adopted a five-point Likert scale (1 = very satisfactory, 5 = very unsatisfactory). The development of these items was based on prior studies (e.g., [78–80]) with corresponding modifications to suit the specific context of this study. We developed the original questionnaire in English and then converted it into Chinese using the method of forward and backward translation, so as to fit the Chinese context. The online survey was carried out on the Wenjuanxing website (https://www.wjx.cn/), one of the largest professional data-collection websites in China, in August, 2022. We invited the colleagues and students to share the link of our online survey on their social media. To ensure the quality of the questionnaire design, we conducted a pre-survey, through which some adjustments were made to the questionnaire.

**Table 1:** The development of items influencing customer satisfaction.

Items	References
Commodity brand building	Elsäßer and Wirtz, 2017 [24]; Dash, Kiefer and Paul, 2021 [25];
Commodity pricing	Morschett, Swoboda and Foscht, 2005 [22]; Zhao et al., 2021 [69]; Yang et al., 2022 [74]; Nilashi et al.,2023 [28]
Authenticity of commodity information	Nilashi et al., 2023 [28]; Tang, Lau and Ho, 2023 [71]
Variety of commodity	Morschett, Swoboda and Foscht, 2005 [22]
Publicity of live-streaming commerce	Li and Hitt, 2008 [21];
Interface design	Ha and Stoel, 2009 [23]; Kim et al., 2009 [73]; Nia and Shokouhyar,2020 [70]; Bobalca et al., 2021 [75];
After-sale guarantee	Nilashi et al., 2023 [28]; Kawa and Zdrenka, 2023 [27]
Customer service	Ha and Stoel, 2009 [23]; Bobalca et al., 2021 [75]; Nilashi et al., 2023 [28]
Degree of promotion	Bobalca et al., 2021 [75]; Zheng et al., 2023 [30]
Live-streaming atmosphere	Ha and Stoel, 2009 [23]
Depth of live-streaming contents	Mao, 2022 [29]
Supervision of live-streaming platforms	Zheng et al., 2023 [30]
Size of live-streaming platforms	Agag and Masry, 2017 [72]
Privacy protection	Ha and Stoel, 2009 [23]; Kim et al., 2009 [73]
Professionalism of live streamers	Zheng et al., 2023 [30]
Commodity packing	Zhao et al., 2021 [69]; Nilashi et al., 2023 [28]
Logistics service	Wu and Dong, 2023 [26]; Kawa and Zdrenka, 2023 [27]

A total of 455 questionnaires were collected, of which 439 were valid. The majority of our respondents are from Zhejiang, where our university is located. With regard to gender, 54% of the respondents are male and 46% are female. In reference to age, the proportion of respondents aged 35–44 is the highest, reaching 29.8% of the total, followed by 20–34 (25.8%), 45–60 (19.5%), under 20 (14.8%) and above 60 (10.2%). Regarding education background, the proportion of junior middle school and below is the highest, reaching 45.7%, followed by senior school (25%), junior college and undergraduate (21.7%) and postgraduate and above (7.5%). That is to way, the education background of 70.7% of the respondents is senior school and below. As for occupations, the distribution of the five occupations is generally even, with each accounting for approximately 20% of the total. Concerning monthly income, under 3000 accounts for the largest proportion, i.e., 40.5%, followed by 3000–5999 (26%), 10,000–20,000 (13%), 6000–9999 (11%) and above 20,000 (9.5%). In other words, the monthly income of 66.5% of the respondents is lower than 6000. Regarding the comprehension of the industry, 57% of the respondents know what the live-streaming commerce of green agricultural products is, while 43% do not. In relation to the channels of purchasing green agricultural products, offline channels account for the highest proportion (37%), followed by traditional online platforms (33%) and live-streaming platforms (27%). See **Table 2** for more information about the sample.

**Table 2:** Basic information of the sample.

Characteristics	Category	Frequency	%
Gender	Male	237	54
	Female	202	46
Age	<20	65	14.8
	20–34	113	25.8
	35–44	131	29.8
	45–60	85	19.5
	>60	45	10.2
Education background	Junior middle school and below	201	45.7
	Senior school	110	25
	Junior college and undergraduate	95	21.7
	Postgraduate and above	33	7.5
Occupations	Professionals (e.g., teachers and doctors)	83	19
	Clerks	92	21
	Students	75	17
	Elementary Occupations (e.g., farmers and miners)	101	23
	Others	88	20
Monthly income (¥)	<3000	178	40.5
	3000–5999	114	26
	6000–9999	48	11
	10,000–20,000	57	13
	>20,000	42	9.5
Understanding the live-streaming commerce of green agricultural products?	Yes	290	57
	No	149	43
Channels of purchasing green agricultural products	Offline channels	162	37
	Traditional online platforms	145	33
	Live-streaming platforms	119	27
	others	13	3

Note: The classification of occupations included in our survey is based on the International Standard Classification of Occupations (ISCO).

### Research methods

This study employs exploratory factor analysis to ascertain the factors determining customer satisfaction in the live-streaming commerce of green agricultural products. Exploratory factor analysis is helpful to analyze the interdependencies among the observed variables and the underlying theoretical constructs, often called factors, in order to detect the underlying structure of observed variables [81]. Based on the results of exploratory factor analysis, this article adopts partial least squares path modeling (PLS-PM) to probe how the identified factors shape customer satisfaction in the live-streaming commerce of green agricultural products. PLS-PM is a multivariate statistical technique using an alternating least squares algorithm, which extends principal component and canonical correlation analysis [82]. In PLS-PM, the direction of the arrows usually indicates which weights are used to develop a construct measurement.

## Empirical results

In this study, SPSS 27.0 is used for exploratory factor analysis and R is employed to construct the PLS-PM model. In exploratory factor analysis, reliability and validity tests are implemented prior to the extraction of the factors. Based on this, PLS-PM is established to study the complete multivariate correlation between explicit variables and latent variables.

### Sample

As shown in **Table 3** the average values of consumers’ evaluation of the factors affecting their satisfaction in the live-streaming commerce of green agricultural products fluctuate between 2.05 and 2.37, with the lowest mean being 2.05 for variety of commodity. This suggests that consumers are satisfied with the variety of commodity provided by live-streaming commerce platforms, which clearly understand the products consumers need at this stage. The factor with the highest mean, i.e. 2.37, is depth of live-streaming contents, followed by authenticity of commodity information, the mean of which is 2.36. In other words, consumers are less satisfied with depth of live-streaming contents and authenticity of commodity information in comparison with the other factors.

**Table 3:** Descriptive statistics.

Items	Factor loadings	Weight	Name	N	Mean	Std. deviation	F1	F2	F3	F4
Degree of promotion	0.790	0.276	Factor of live-streaming contents	168	2.20	0.89	0.790			
Live-streaming atmosphere	0.794	0.277		168	2.22	0.91	0.794			
Professionalism of live streamers	0.654	0.229		168	2.37	0.97	0.654			
Depth of live-streaming contents	0.624	0.218		168	2.31	0.95	0.624			
Privacy protection	0.842	0.232	Factor of supporting services	168	2.28	0.97		0.842		
Commodity packing	0.793	0.219		168	2.20	0.87		0.793		
Logistics service	0.719	0.198		168	2.19	0.88		0.719		
After-sale guarantees	0.648	0.179		168	2.35	1.04		0.648		
Customer service	0.625	0.172		168	2.25	0.99		0.625		
Supervision of live-streaming platforms	0.714	0.238	Factor of live-streaming platforms	168	2.31	0.95			0.714	
Size of live-streaming platforms	0.738	0.246		168	2.24	0.90			0.738	
Publicity of live-streaming commerce	0.769	0.256		168	2.13	0.87			0.769	
Interface design	0.776	0.259		168	2.22	0.96			0.776	
Commodity brand building	0.850	0.307	Factor of commodity	168	2.23	0.94				0.850
Commodity pricing	0.616	0.223		168	2.15	0.86				0.616
Authenticity of commodity information	0.553	0.200		168	2.36	1.00				0.553
Variety of commodity	0.751	0.271		168	2.05	0.84				0.751

### Exploratory factor analysis

Prior to exploratory factor analysis, reliability and validity tests were carried out. The coefficient of Cronbach’s alpha is 0.996, approximating 1, denoting the high reliability of the sample [83]. The KMO test and Bartlett’s test of sphericity were used to determine whether factor analysis was applicable to the data. The KMO is 0.94, approximating 1, indicating the high validity of the sample, to which factor analysis is applicable [84]. At the same time, the P value of the Bartlett sphericity test is 0.00, smaller than 0.05, suggesting a correlation between items, to which factor analysis is applicable. Since the slope of the scree plot begins to flatten out in the fifth factor, indicating a weakened ability to explain variance, we decided to extract four principal components in total. The "Initial eigenvalues" of components 1–4 are greater than 0.6, denoting a large contribution, and the corresponding "Cumulative" reached 81.754%, indicating a strong ability to explain variance, therefore the requirements of factor analysis are satisfied. Based on the size of their loadings, we categorized the items into four main factors (see **Table 3**). The first factor has large loadings in the following four items: commodity brand building, commodity pricing, authenticity of commodity information and variety of commodity, which are related to the commodity. Thus, we termed the first factor, i.e., F1, the factor of commodity. The second factor has large loadings in the following four items: supervision of live-streaming platforms, size of live-streaming platforms, publicity of live-streaming commerce and interface design, which are related to the live-streaming platforms. Therefore, we termed the second factor, i.e., F2, the factor of live-streaming platforms. The third factor has large loadings in the following four items: degree of promotion, live-streaming atmosphere, professionalism of live-streamers and depth of live-streaming contents, which are related to the live-streaming contents. Consequently, we termed the third factor, i.e., F3, the factor of live-streaming contents. The fourth factor has large loadings in the following five items: privacy protection, commodity packing, logistics service, after-sale guarantees and customer service, which are related to the supporting services. Hence, we term the fourth factor, i.e., F4, the factor of supporting services.

As **Table 3** demonstrates, "commodity brand building" has the highest loading for the factor of commodity, "interface design" has the highest loading for the factor of live-streaming platforms, "live-streaming atmosphere" has the highest loading for the factor of live-streaming contents and "privacy protection" has the highest loading for the factor of supporting services. In other words, successful commodity brand building, comfortable interface design, good live-streaming atmosphere and necessary privacy protection play important roles in customer satisfaction with the live-streaming commerce of green agricultural products.

In order to measure discriminant validity, we employed the Heterotrait-Monotrait ratio (HTMT) and the criterion of Fornell and Larcker (1981). Based on Fornell and Larcker [85], each factor should show a stronger relationship with its own factor compared to other factors for satisfactory discriminant validity. According to **Table 4**, each of the diagonal elements, i.e., the square root of the AVE between the factors, is larger compared to the non-diagonal elements, i.e., relationships between factors, suggesting acceptable discriminant validity. Meanwhile, all values of HTMT are below 0.90, denoting satisfactory discriminant validity [86]. Founded on the above analysis, the items manifest reliability and sufficient discriminant validity.

**Table 4:** The measurements’ discriminant validity.

Fornell-Larcker criterion	Factor of live-streaming contents	Factor of supporting services	Factor of live-streaming platforms	Factor of commodity
Factor of live-streaming contents	0.845			
Factor of supporting services	0.811	0.855		
Factor of live-streaming platforms	0.635	0.656	0.688	
Factor of commodity	0.504	0.397	0.205	0.585
HTMT Criterion	Factor of live-streaming contents	Factor of supporting services	Factor of live-streaming platforms	Factor of commodity
Factor of live-streaming contents				
Factor of supporting services	0.876			
Factor of live-streaming platforms	0.751	0.769		
Factor of commodity	0.654	0.499	0.284	

### Partial least squares path modeling (PLS-PM)

**Fig 1:**
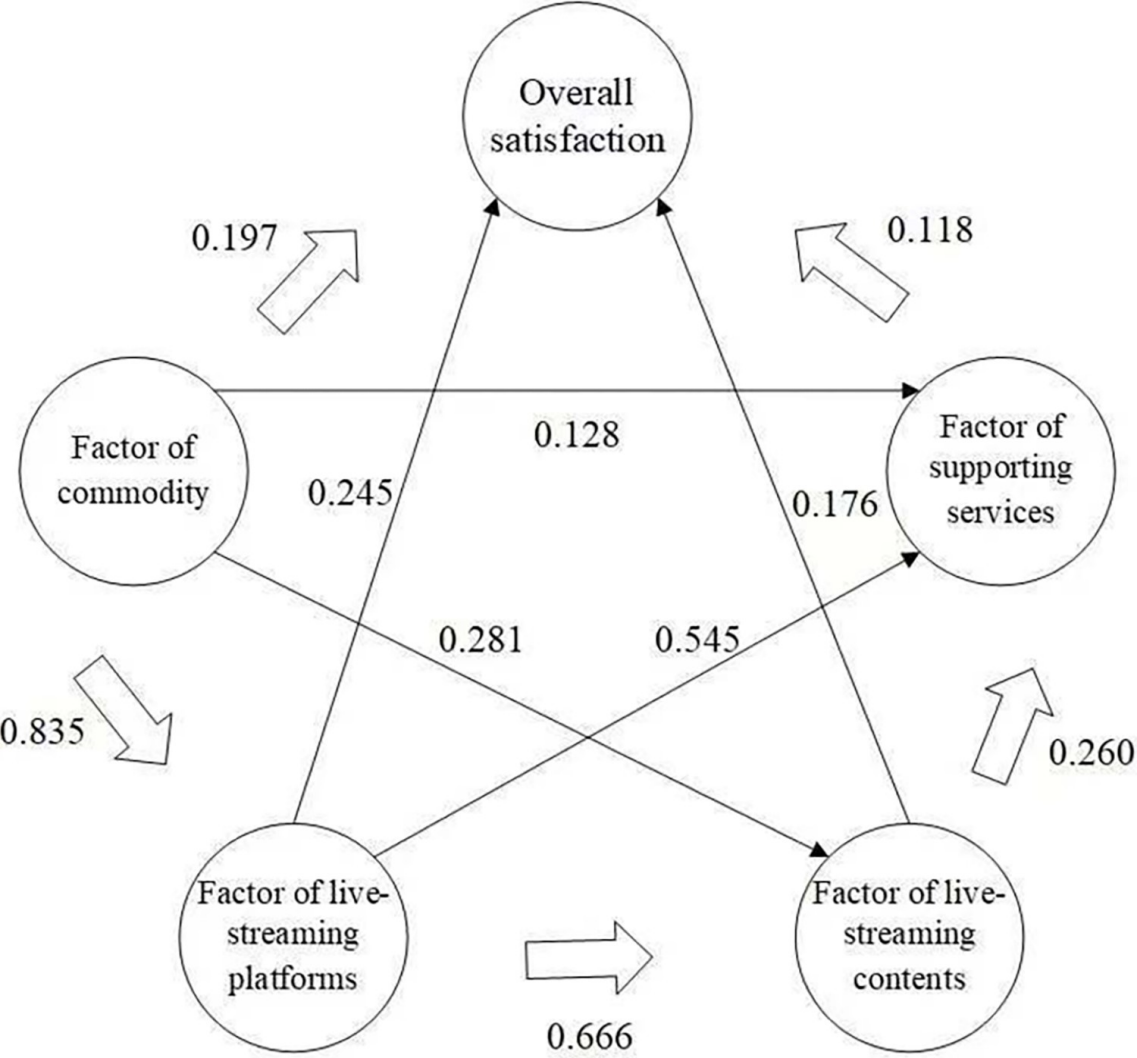
PLS-PM model.

Through exploratory factor analysis, this article categorizes the 17 items, which influence customer satisfaction in the live-streaming commerce of green agricultural products, into four factors, namely factor of commodity, factor of live-streaming platforms, factor of live-streaming contents and factor of supporting services. In order to further explore the relationship between these factors and consumers’ overall satisfaction, we developed a PLS-PM model, as shown in **Fig 1**.

After establishing the path modeling graph, reliability test (see **Table 5**) and goodness-of-fit test (see **Table 6**) were implemented for the PLS-PM. As **Table 5** demonstrates, the Cronbach’s alpha coefficients of the five factors are all greater than 0.85, the values of Dillon-Goldstein’s rho are all greater than 0.9, the first eigenvalues are all greater than 1 and the second eigenvalues are all smaller than 1, indicating the good reliability of the model (e.g., [87, 88]). As shown in **Table 6**, all communalities are greater than 0.5 and the values of average variance extracted (AVE) range from 0.695 to 1.000, indicating the good convergent validity of the items [85].

**Table 5:** Reliability test of PLS-PM model.

Items	Mode	MVs	Cronbach’s alpha	Dillon-Goldstein’s rho	First eigenvalue	Second eigenvalue
Factor of commodity	A	4	0.878	0.916	2.93	0.448
Factor of live-streaming platforms	A	4	0.853	0.901	2.78	0.600
Factor of live-streaming contents	A	4	0.902	0.932	3.09	0.353
Factor of supporting services	A	5	0.932	0.948	3.93	0.442
Overall satisfaction	A	1	1.000	1.000	1.00	0.000

**Table 6:** Goodness of fit test of PLS-PM model.

Items	Type	*R* ^2^	Communalities	AVE
Factor of commodity	Exogenous	0.000	0.731	0.731
Factor of live-streaming contents	Exogenous	0.836	0.772	0.772
Factor of live-streaming platforms	Exogenous	0.697	0.695	0.695
Factor of supporting services	Exogenous	0.810	0.783	0.783
Overall satisfaction	Exogenous	0.484	1.000	1.000

The values of *R*^2^ are basically greater than 0.6, indicating that the model has certain predictive capability [82]. Some unsatisfactory results, i.e. *R*^2^ for factor of commodity and overall satisfaction, may be related to our processing of the sample data. Nevertheless, the overall predictive capacity of the model is basically acceptable.

According to the results in **Table 7**, all factors, i.e. factor of commodity, factor of live-streaming platforms, factor of live-streaming contents and factor of supporting services, have an impact on the overall satisfaction. On the one hand, the factor of live-streaming platforms has the greatest direct impact on the overall satisfaction, reaching 0.245. On the other hand, the factor of commodity has the strongest indirect influence, i.e., 0.446, and the strongest total influence, i.e., 0.643, on the overall satisfaction among all the four factors. Meanwhile, it also has a substantial direct influence on the other factors, i.e., factor of live-streaming platforms (0.835), factor of live-streaming contents (0.281) and factor of supporting services (0.128). In addition, the factor of live-streaming platforms has the strongest direct impact on the factor of live-streaming contents, reaching 0.666. Furthermore, all the other factors, i.e., factor of commodity (0.128), factor of live-streaming platforms (0.545) and factor of live-streaming contents (0.260), influence the factor of supporting services to some degree.

**Table 7:** PLS-PM model effect.

Path	Direct effect	Indirect effect	Total effect
Factor of commodity-> factor of live-streaming contents	0.281	0.556	0.837
Factor of commodity-> factor of live-streaming platforms	0.835	0.000	0.835
Factor of commodity-> factor of supporting services	0.128	0.673	0.801
Factor of commodity-> overall satisfaction	0.197	0.446	0.643
Factor of live-streaming platforms-> factor of live-streaming contents	0.666	0.000	0.666
Factor of live-streaming platforms-> factor of supporting services	0.545	0.174	0.719
Factor of live-streaming platforms-> overall satisfaction	0.245	0.202	0.447
Factor of live-streaming contents-> factor of supporting services	0.260	0.000	0.260
Factor of live-streaming contents-> overall satisfaction	0.176	0.031	0.207
Factor of supporting services -> overall satisfaction	0.118	0.000	0.118

## Discussion

Under the circumstances of excessive resource consumption, severe environmental pollution and a rising demand for green agricultural products, it is necessary for agriculture to follow a green development route. An important way to realize the green revolution of agriculture is to raise the long-term benefits of green agriculture for farmers [89]. Therefore, it is essential to promote the sale of green agricultural products for the sustainable development of agriculture. For consumers, besides price, inadequate comprehension and inconvenient purchase channels result in the low market share of green agricultural products [90]. Since the combination of online and offline channels can accelerate the circulation of agricultural products in the process of trading, consumers are increasingly using e-commerce to purchase green agricultural products. Over time, consumers have become more demanding as they have better understanding of their purchasing power [2]. Within such a context, it is vital for vendors to satisfy consumers’ demands. Given that live-streaming commerce, a subset of e-commerce that incorporates real-time social interaction, provides an effective new channel to promote sales, this article explores the antecedents of customer satisfaction in the live-streaming commerce of green agricultural products. The main findings are as follows.

First, most consumers are satisfied with the live-streaming commerce of green agricultural products and the degree of their overall satisfaction is medium to high, implying that there are still problems in the live-streaming commerce of green agricultural products that need to be improved. Second, by virtue of exploratory factor analysis, we extracted four antecedents of customer satisfaction in the live-streaming commerce of green agricultural products, i.e., commodity, live-streaming platforms, live-streaming contents and supporting services. Among the variables related to commodity, "commodity brand building" had the highest weight. As a guarantee of quality, the brand increases the confidence of customers in their expectations being satisfied [91]. Furthermore, customer loyalty, the prerequisite of which is customer satisfaction, can only be achieved when consumers are aware of the significant differences between competing brands (e.g., [92, 93]). Thus, it is not surprising that "commodity brand building" had the highest weight among the variables relevant to the factor of commodity. Among the variables related to live-streaming platforms, "interface design" had the highest weight. Sohn et al. [94] illustrated that the more complex the interface design of a shopping website, the higher the chance that consumers feel visually crowded when searching for products online, hence decreasing the visual appeal and satisfaction. As a consequence, it is reasonable that "interface design" had the highest weight among the variables associated with the factor of live-streaming platforms. Among the variables related to live-streaming contents, "live-streaming atmosphere" had the highest weight. The uniqueness of live streaming lies in the fact that consumers can interact with sellers in real time, leading to an immersive, engaging shopping experience and a more interpersonal association (e.g., [95, 96]). Specifically, the interactive communication between streamers and consumers creates an interactive feedback signal to consumers, which can then generate a powerful psychological hint to consumers and enhance their trust in the streamers. In other words, the interactive atmosphere strengthens the communication between sellers and buyers and then facilitates the satisfaction of consumers in live-streaming commerce. Therefore, it is legitimate that "live-streaming atmosphere" had the highest weight among the variables related to the factor of live-streaming contents. Among the variables related to supporting services, "privacy protection" had the highest weight. As the daily usage of social media continues to grow, privacy concerns are increasing as a huge amount of identifiable consumer information is archived, accumulated and connected across different social media platforms [97]. Undoubtedly, consumers’ concerns about privacy negatively influence their willingness to use e-commerce services [98]. Thus, it is logical that "privacy protection" had the highest weight among the variables pertinent to the factor of supporting services. In short, commodity brand building, a good live-streaming atmosphere, clear interface design and necessary privacy protection play the most important roles in customer satisfaction with the live-streaming commerce of green agricultural products.

Third, by virtue of a PLS-PM model, we found that commodity, live-streaming platforms, live-streaming contents and supporting services had a significant positive impact on consumers’ overall satisfaction. Specifically, the direct effect of the live-streaming platforms on overall satisfaction was the strongest, reaching 0.245, while supporting services had the weakest direct effect on overall satisfaction at only 0.118. At the same time, commodity had the strongest indirect effect on overall satisfaction, reaching 0.446, and its total effect on overall satisfaction was also the strongest, reaching 0.643. In addition, there were also interactions between different factors, e.g., the direct effect of commodity on live-streaming platforms was the strongest at 0.835, while the direct effect of commodity on supporting services was the weakest at 0.128. Consistent with Park and Lin [40], who indicated that consumers’ favorable perceptions of commodity and live-streaming content enhance their purchase intention, this study found that the more positive the consumers’ attitude toward commodity and live-streaming content, the higher their satisfaction with live-streaming commerce. We also observed that the good development of live-streaming platforms can improve customer satisfaction in live-streaming commerce. This finding is consistent with Lu and Chen [99], who found that improving the live-streaming platforms positively affects consumers’ purchase intention. Meanwhile, our study reveals that supporting services are positively correlated with customer satisfaction. This finding is consistent with prior studies which demonstrated that factors related to supporting services play a significant role in the consumer experience in e-commerce (e.g., [30, 100]).

## Conclusions and implications

In summary, our study indicates that the majority of consumers involved in live-streaming commerce for green agricultural products report a medium to high overall satisfaction degree, suggesting that the integration of live-streaming technology has generally been successful in promoting the sale of these products. Meanwhile, this research identifies four key antecedents influencing customer satisfaction in the context of live-streaming commerce for green agricultural products, including commodity-related factors, attributes of live-streaming platforms, characteristics of live-streaming contents and the quality of supporting services. Besides, "Commodity brand building" emerges as a crucial factor with the highest weight among the commodity-related variables, underscoring the importance of investing in brand development for green agricultural products to positively impact customer satisfaction. Therefore, businesses should focus on establishing strong and recognizable brands to enhance consumer perception. Moreover, our study identifies specific variables within live-streaming platforms, live-streaming contents, and supporting services that significantly contribute to customer satisfaction, including "interface design" for platforms, "live-streaming atmosphere" for contents and "privacy protection" for supporting services. These findings offer practical insights for businesses looking to optimize their offerings in these areas. Furthermore, live-streaming platforms exert the strongest direct influence on customer satisfaction among the antecedents, suggesting that businesses should prioritize investments and improvements in platform-related features, functionalities and user experience to enhance overall customer satisfaction. In addition, while live-streaming platforms have the strongest direct impact, the commodity category has the strongest indirect and total influence on customer satisfaction, implying that while platform features directly impact satisfaction, the overall quality and perception of the green agricultural products indirectly and collectively contribute significantly to customer satisfaction.

### Theoretical implications

Theoretically, this article contributes to past research on e-commerce by being one of the first empirical studies conducted under the live-streaming context, which considerably modifies the way online sellers communicate with consumers and sell their products. We extended prior studies on the characteristics of live-streaming commerce and the motivation of consumers to participate by investigating the antecedents of customer satisfaction in the live-streaming commerce of green agricultural products. Meanwhile, we also offered empirical evidence for the validity of the SERVQUAL model in the context of live-streaming commerce of green agricultural products, a seldom tapped topic in prior studies, by founding our research on the integration of EDT and the SERVQUAL model. Furthermore, we enriched academic endeavors into the antecedents of customer satisfaction using a comprehensive theoretical model which integrates items explored separately in past research. Specifically, we shed light on the topic of online customer satisfaction, with particular relevance to the field of consumer psychology and digital marketing. We have, in effect, combined the perspectives of commodity, live-streaming platforms, live-streaming contents and supporting services and applied them to the realm of consumer psychology in a way which is of significance for consumer behavior and marketing in the context of live-streaming. For example, given that customer satisfaction is key to the success of a company and that its antecedents in the live-streaming context are uncertain, this study ascertains four factors that influence customer satisfaction in the live-streaming commerce of green agricultural products, namely commodity, live-streaming platforms, live-streaming contents and supporting services.

### Managerial implications

From a managerial viewpoint, our study provides insights into how online sellers can employ live-streaming technology to increase customer satisfaction by ascertaining its antecedents and the routes along which they influence customer satisfaction. Since the identification and utilization of the factors that determine customer satisfaction could be an essential competitive advantage [101], our findings are significant for the sustainable development of the company and the industry, i.e. the live-streaming commerce of green agricultural products. The highest weight attributed to "commodity brand building" suggests that businesses should prioritize and invest in building strong and recognizable brands for their green agricultural products, e.g., through effective marketing strategies, emphasizing quality, sustainability and unique selling propositions. Given that live-streaming platforms have the strongest direct influence on customer satisfaction, businesses should focus on improving the design and functionality of their platforms, e.g., optimizing the user interface, ensuring a seamless and engaging live-streaming atmosphere and paying attention to privacy protection features to enhance the overall user experience. Recognizing the importance of "live-streaming contents," businesses should prioritize the creation of high-quality, informative and engaging content, e.g., showcasing the agricultural products, explaining their origins and providing relevant information to capture and retain the audience’s interest. Recognizing the multi-faceted nature of customer satisfaction in live-streaming commerce, businesses should adopt a holistic approach that considers the interplay between commodities, live-streaming platforms, contents, and supporting services. Strategic planning should involve balancing investments across these factors to achieve comprehensive customer satisfaction. The variable "privacy protection" within supporting services highlights the significance of privacy concerns for customers. Businesses should implement robust privacy protection measures and communicate them transparently to build trust among consumers. Additionally, ensuring excellent customer support and addressing any concerns promptly can contribute to overall customer satisfaction. The medium to high overall satisfaction degree suggests that the current strategies are generally effective. However, businesses should continuously monitor customer feedback, adapt strategies based on evolving consumer preferences, and stay attuned to market trends to maintain and enhance customer satisfaction over time.

### Limitations and future research

Although it provides implications for both academia and the live-streaming industry, this study is not without its limitations. First, the education background is senior school and the monthly income is less than 6000 for about 70% of the respondents. In other words, about 2/3 of the respondents are undereducated low-income consumers, indicating the narrow coverage of the sample. This may, in turn, cast doubt on whether our findings still hold when applied to different customer segments, e.g., those with high income. Therefore, future research should investigate customers with different characteristics, such as education background, income level, product knowledge and online shopping involvement. Second, we only took green agricultural products into consideration when exploring the antecedents of customer satisfaction in live-streaming commerce. However, Zhang et al. [102] found that product category has a significant influence on consumers’ purchase intention, which is closely related to their satisfaction, in live-streaming commerce. Consequently, it would be fruitful to explore in more detail the interplay between customer satisfaction and product category in future studies.
